# Operative Surgery, Based on Normal and Pathological Anatomy

**Published:** 1852-01

**Authors:** 


Art. V.—*Operative Surgery, based on Normal and Pathological Anatomy.* By J. F. Malgaigne, Professor Agregé de la Faculté de Medicine de Paris, Chirurgien de l' Hopital de Lourcine, Chevalier de la Legion d' Honneur, et du Mente Militaire de Pologne, etc., etc. Translated from the French, by Frederick Brittan, A. B., M. D., M. R. C. S- L. Illustrated by wood engravings, from designs, by Dr. Westmacott. Philadelphia: Blanchard & Lea: 1851. pp. 565: 8 vo.

The original term for the surgical art meant literally "operative surgery," but in our day the term has come to have a much more comprehensive signification, and implies not only the *chirurgery* of the ancients, but surgical pathology. By our French brethren the phrase "medicine operatoire" proposed by Sabatier, is the one employed to designate this branch of the profession, but they embrace in it only a part of operative surgery, arbitrarily excluding many things which we range under it, e. g. the application of bandages, and the reduction of luxations and fractures. In this they act less wisely than the surgeons of England and America, but at the same time let us admit that both in historical and operative surgery, they have hardly any equals in the world. With them much more than with any other nation, the history of surgery is a study; and Velpeau, following in the path which Sabatier first opened, has excelled all his contemporaries in this line of labor. In a rapid sketch of the progress of surgery, Malgaigne mentions "a deficiency of anatomical data" as one of the circumstances by which its march has been retarded, and relates the strange fact, that when the Faculty of Medicine of Paris wished, in the beginning of the last century, to institute a course for the repetition of operations on the dead subject, the whole corporation of surgeons rose with great clamor against this innovation, which it denounced as rash and fatal. The candid Frenchman admits, that *medicine operatoire* in England, moulded on pathological anatomy and experiment, after the example and lessons of John Hunter, "for a moment surpassed" French operative surgery, and even now is almost its equal, so vigorous was the impulse it received from the hand of that great master. But in neither country does he believe the art has reached perfection, or accomplished all that it ought to do. Especially among his own countrymen, he is aware that it has not always taken the best direction.

"Perhaps," he says, "in the real progress made in our time by *Medicine Operatoire*, its end has been overlooked: whilst practicing the operations on the dead body, perhaps the living patient has been too much forgotten; whilst perfecting the operations, all that should precede and follow them—the indications and results—have been left in the shade. In the first editions of this book, I had stated, "A treatise on operative surgery, to satisfy all the requirements of the age, should, for each operation,—first, discuss the indications, exactly study the surgical anatomy, review all the proceedings, and, after mature examination and judicious choice of the best, describe the manipulation with all the necessary detail;—then point out the different methods of dressing; set forth a statistical account of successes and failures; and, lastly, seek in post-mortem appearances the causes of death in fatal cases, in order to point out their remedy." But at the present day even these conditions no longer suffice. Experience teaches us daily that we must not regard as completely cured all those who apparently recover; and that it is extremely necessary to keep an exact account of the relapses, both in relation to the nature of the disease, and according to the proceeding adopted. This is not all; for, after the most positive cure, it is still interesting to study the consequences of each operation, as well on the organs and functions, as on the general vitality of the patient. Any observation that does not extend so far should be considered incomplete. This is almost a new field offering itself to the surgeons of the present day."

In his work he has not attempted to give even an outline of the history of the several operations embraced in it, but has described each in a succinct but clear and intelligible manner, giving the results without discussion.—The parts which he has treated with especial care, are the surgical anatomy and the operative manipulations, precisely the points upon which the young surgeon is desirous of information, and it is doubtless the excellence of his manual in this respect that has given it such currency in all the countries of Europe. It is just such a guide as the operator wants, embracing every case likely to demand his interference. In the time since it was published, (1842,) operative surgery has received a great boon to which it makes no reference; no mention is made among the means of diminishing pain during operations, of the discovery of our modern anesthetic agents; but this is a deficiency which every young surgeon in our country can readily supply.

The analysis of a work such as this, is a matter out of the question. In its composition it was the study of the author to condense his matter as much as possible, and any farther compression would render its details unintelligible. It is a work not only to be diligently perused by the student, but to be consulted continually by the practical surgeon, whose mind requires to be refreshed on any of the procedures of his art. The teachings of Malgaigne are not always such as the experienced surgeon will adopt, as, indeed, what writer's are? As to the best mode of performing most of the operations, there is a diversity of opinions among practitioners, and the operator is obliged to decide in each case for himself; but, in the main, our impression is, that no safer guide has yet been offered to the acceptance of the profession than the volume of which we thus take our leave.

